# Configurational Setup for Fully Robotic Distal Pancreatectomy with Splenectomy Using Hugo™ RAS and Ligasure™ RAS Maryland: First Case Report (With Video)

**DOI:** 10.3390/jcm15062423

**Published:** 2026-03-22

**Authors:** Orlin Belyaev, Hussein Salama, Tim Fahlbusch, Waldemar Uhl

**Affiliations:** Department of General and Visceral Surgery, St. Josef Hospital, Ruhr University Bochum, Gudrunstr. 56, 44791 Bochum, Germany; hussein.salama@kklbo.de (H.S.); tim.fahlbusch@kklbo.de (T.F.); waldemar.uhl@kklbo.de (W.U.)

**Keywords:** robotic surgery, distal pancreatectomy, splenectomy, Hugo™ RAS, Ligasure™ RAS

## Abstract

**Background/Objectives:** Fully robotic pancreatic resections using the Hugo™ RAS platform have not yet been described in the literature. **Methods:** A 72-year-old male with a cystic lesion in the pancreatic tail underwent a fully robotic distal pancreatectomy and splenectomy using the Hugo RAS platform and the newly introduced robotic vessel sealer LigaSure RAS. The proposed configurational setup and technical details are described. **Results:** The procedure was completed safely without complications: blood loss was <50 mL, total duration of surgery was 305 min, and console time was 195 min. The postoperative period was uneventful, and the patient was discharged on postoperative day 7. **Conclusions:** Distal pancreatectomy with the Hugo RAS platform may be feasible and safe in selected cases.

## 1. Introduction

Robotic distal pancreatectomy (RDP) has increasingly been performed over the last decade, especially at dedicated high-volume pancreatic centers [1]. Reported advantages include lower conversion and infection rates, reduced blood loss, and shorter hospital stays at the price of longer operative times and costs, compared to open (ODP) and laparoscopic (LDP) surgery [2]. Further, RDP significantly reduces the ergonomic stress, procedural effort and frustration, visual strain and cognitive overload of the surgeon compared to LDP [3]. For over two decades, the da Vinci™ Surgical System (Intuitive Surgical Inc., Sunnyvale, CA, USA) has been the only available robotic platform in the field of general and hepato-pancreato-biliary (HPB) surgery. Lately, several new surgical robot platforms for clinical practice have become available. The Toumai robotic surgical platform (MicroPort^®^MedBot™ Group Company, Shanghai, China), the MP series robotic platform (Edge Medical Company, Shenzhen, China) as well as the hinotori™ Surgical Robot System (Medicaroid Corporation, Kobe, Japan) have been used in RDP with success [4,5]. While all these platforms share a da Vinci-like single cart architecture, the Hugo™ RAS System (Medtronic, Dublin, Ireland) offers an open console combined with a modular design of four independent arm carts. It was CE-certified for general surgery in late 2022. Due to the initial limited choice of instruments and lack of experience, early reports on its feasible and safe use focused on hernia repair and bariatric and colorectal surgery [6]. Recently, Nagai et al. reported of having performed the first-ever Hugo RAS-assisted RDP [7]. However, key steps of the procedure were laparoscopically done by the bed assistant using a laparoscopic LigaSure Maryland device, including opening the bursa, mobilization of the splenic flexure and vessel sealing and dissection. Hugo RAS was introduced at our institution in 2022, and we had the opportunity to perform the first general surgical procedures in Germany. Since then, our team has gathered pioneering experience of over 350 Hugo procedures, which allowed us to establish our own standard setups for both upper and lower gastrointestinal surgery [8]. At the same time, with an annual volume of about 400 pancreatic procedures, our institution is one of the largest certified pancreatic centers in Germany. This experience and the recent market approval of the newly developed LigaSure™ RAS Maryland by Medtronic offer excellent prerequisites for performing safe pancreatic surgery with the Hugo RAS platform. Nevertheless, the use of Hugo RAS for pancreatic surgery is still off label, since there is no official certification for HPB surgery by the EMA (European Medicines Agency) or the FDA (U.S. Food and Drug Administration).

This article aims to describe the configurational setup and technical details of a fully robotic RDP using the Hugo RAS platform and the newly developed LigaSure™ RAS Maryland vessel sealing device.

## 2. Materials and Methods

### 2.1. Patient

The patient was a 72-year-old man with a body mass index of 25 kg/m^2^ (184 cm, 85 kg) referred to our hospital for evaluation of a cystic lesion of the pancreatic body, incidentally detected on a computed tomography (CT) scan 2 years ago during evaluation of an ulcer of the aortic arch. The patient suffered intermittent episodes of nausea and postprandial epigastric pain, accompanied by 10 kg weight loss over the last 6 months. The lesion grew from 10 mm to 22 mm over 2 years and developed solid enhancing components, seen on CT, magnetic resonance cholangio-pancreaticography (MRCP) and on endoscopic ultrasonography (Table 1).

**Figure 1:**
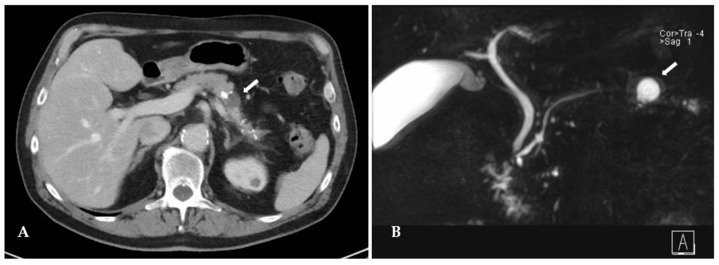
Preoperative CT (**A**) and MRCP (**B**) images of a cystic lesion (white arrow) in the pancreatic body.

Calcifications were present just proximal to the cyst as well as in the pancreatic tail distal to it, which were not present in the pancreatic head (Figure 1). There was no history of alcohol abuse, hypercalcemia; however, the patient admitted having been an active chain smoker for over 40 years. Diabetes mellitus was diagnosed two years ago. Tumor markers CA19-9 and CEA were within the normal range. The lesion was suspected to be either a side-branch intraductal papillary mucinous neoplasia (IPMN) with worrisome features or a pseudocyst in chronic pancreatic tail pancreatitis. Because of a positive family history of pancreatic cancer, accumulation of symptoms and development of worrisome imaging features, the patient was scheduled for a distal pancreatectomy with splenectomy and written informed consent was obtained.

### 2.2. Surgical Setup

The patient was placed in the lithotomy position. Access to the abdomen was achieved in Hasson’s open technique through a vertical incision in the middle line 3 cm above the umbilicus. An 11 mm port was inserted and CO_2_ insufflated to a pressure of 12 mmHg. After inspection of the abdominal cavity, an 8 mm port was inserted 2 cm below the left costal arch, 16 cm apart from the first one; then, a second 11 mm port was inserted 3 cm caudal to the midpoint of the first two ports. A second 8 mm port was placed in the epigastric area just to the left of the middle line, 15 cm above the umbilicus. A 12 mm assistant port (Endopath XCEL™, J&J Medtech, New Brunswick, NJ, USA) was placed in the right upper abdomen at the level of the first trocar and 8 cm apart from it.

### 2.3. Surgical Procedure

**Figure 2:**
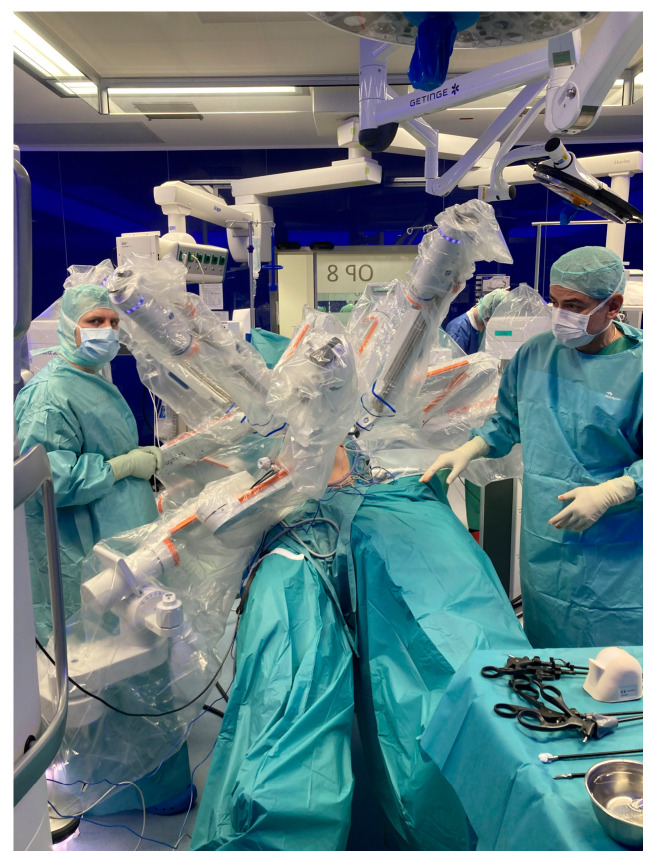
Operating room with docked robotic arms of the Hugo RAS.

A DP was performed in the clockwise manner in two robotic steps needing one redocking of the system. Both steps were performed using the so-called butterfly configuration with two arms on each side of the patient (Figure 2).

**Figure 3:**
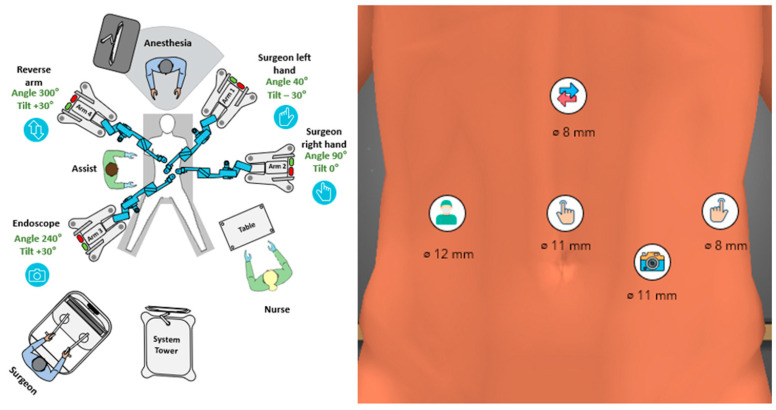
Configurational setup for step 1 of RDP with the Hugo RAS.

For step 1, the table was tilted 20° head-up and 20° left-side-up. Arm 1 was appointed to the surgeon’s left hand and carried the bipolar grasper, while arm 2 was appointed to the right hand carrying either the curved monopolar shears or the Ligasure RAS vessel sealer. Arm 3 was connected to the endoscope and arm 4 carried the double-fenestrated grasper appointed also to the left hand of the surgeon (Figure 3). During step 1, wide mobilization of the splenic flexure, opening of the omental bursa and division of the short gastric vessels were performed. This allowed an excellent view of the pancreatic body and tail and assessment of the lesion.

**Figure 4:**
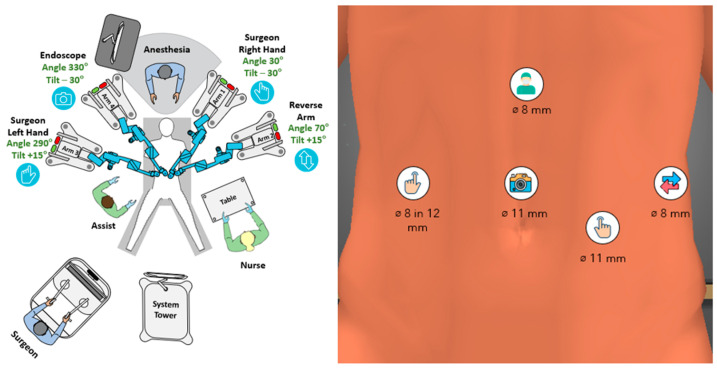
Configurational setup for step 2 of RDP with the Hugo RAS.

Redocking for step 2: The table tilt was reduced to 10° head-up and 10° left-side-up. An 8 mm robotic port was inserted in the 12 mm assist port, connected to arm 3 and appointed to the left hand of the surgeon carrying either the bipolar grasper or the Ligasure RAS at a later stage. The endoscope was moved to the supraumbilical port and connected to arm 4. Arm 1 was appointed to the right hand carrying the monopolar shears/Ligasure RAS and arm 2 carried the double-fenestrated grasper as a reverse second right hand to retract the pancreas (Figure 4).

**Figure 5:**
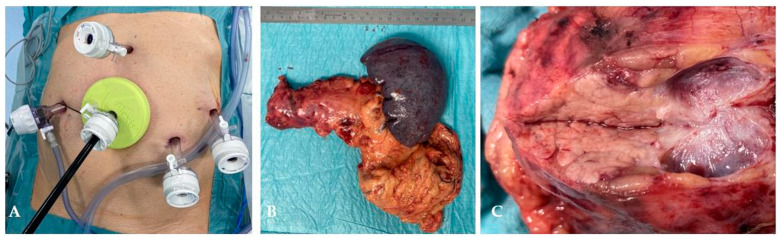
View of ports and wound retractor during specimen extraction (**A**); specimen consisting of pancreatic tail, spleen and omentum (**B**); cut surface of the cystic lesion (**C**).

During step 2, the splenic vessels were dissected free under the pancreas; then, the splenic artery was divided using an Endo GIA Ultra 30 mm white cartridge (Medtronic, Minneapolis, MN, USA) and the splenic vein was divided in the same manner, preserving the left gastric and inferior mesenteric veins. The pancreas was divided at the level of its isthmus using a purple cartridge Endo GIA Ultra 60 mm after compressing it for 3 min. The pancreatic stump was temporarily covered with a small Tabotamp fleece (Ethicon, J&J, Raritan, NJ, USA) and a gauze. After that, the pancreatic tail with the spleen was dissected free off the retroperitoneum (Supplementary Video S1). Robotic arms were undocked, the supraumbilical incision was increased to 5 cm and a small Alexis wound retractor with a cap was inserted. The specimen was retrieved in an Endo Bag (Medtronic, Minneapolis, MN, USA) through the Alexis retractor (Applied Medical, Rancho Santa Margarita, CA, USA) (Figure 5). It was then covered again for laparoscopic control of the pancreatic stump, covering it with a Tachsoil fleece (Corza Medical, Düsseldorf, Germany), irrigation and drain placement.

## 3. Results

**Figure 6:**
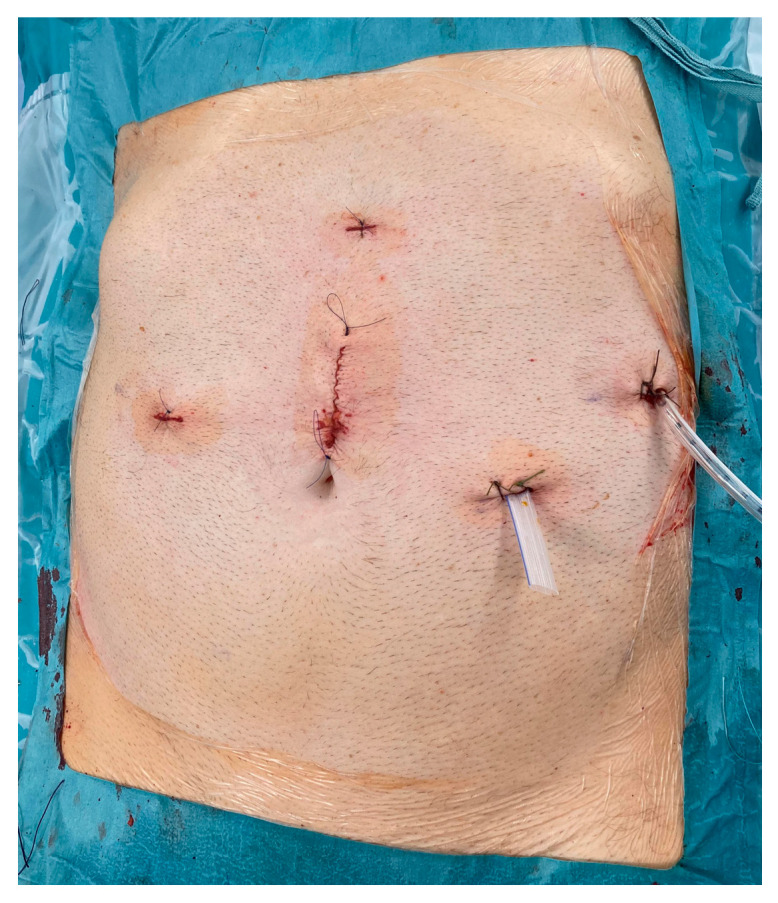
Final view of the abdomen with inserted drains at the end of surgery.

The frozen section confirmed an R0 resection of the lesion and found no malignancy. On final histology, a pseudocyst and chronic pancreatitis in the pancreatic tail were reported; no IPMN was detected. The total duration of surgery was 305 min and the console time was 195 min. Blood loss was less than 50 mL. No device malfunction or arm collisions occurred. There were no perioperative complications; the drains were tested negative for amylase and lipase at days 3 and 5 and consequently removed (Figure 6). The patient recovered quickly and was discharged on postoperative day 7. At first follow-up, 30 days after surgery, a physical examination, abdominal ultrasound and laboratory tests were performed without any pathologic findings. In particular, no signs of wound infection, pleural effusions or fluid collection at the pancreatic stump were found; leukocyte count, CRP, hemoglobin, serum amylase and lipase, liver enzymes and creatinine were within normal limits. The patient was satisfied with the results, completely symptom-free and reported of a much better control of his blood glucose levels compared to the time before surgery.

## 4. Discussion

A wide range of surgical procedures with the Hugo RAS platform have been reported in the literature since the introduction of this novel robotic platform in 2022. After initial experience in urology and gynecology, the volume of evidence in the field of general surgery has recently been growing [6,7,8]. The authors of this case report have successfully performed over 350 general surgical procedures with the Hugo RAS platform, including gastric and upper-GI tumor resections, splenectomies, adrenalectomies, fundoplications, deroofing of liver cysts, cholecystectomies, hernia repairs and the whole spectrum of colorectal resections for benign and malignant disease. At the same time, the team has extensive experience in pancreatic surgery with more than 7000 procedures over the last 20 years and our institution is certified as a high-volume pancreatic center by the German Society of General and Visceral Surgery.

Hugo RAS is still a novel robotic platform, characterized by an open console offering ergonomic and communication advantages for the surgical team. The second specific feature of the system is its modular design with four independent arm carts, which can be positioned virtually anywhere around the table, allowing extreme flexibility and freedom of port and instrument positioning. The most important advantage of Hugo lies in the opportunity to adapt its configuration to the specific characteristics of the patient’s body, the targeted organ and the individual preferences of the surgeon. The drawbacks of the platform include a currently limited choice of instruments, lacking irrigation-suction, clipping and stapling devices. The first-generation Ligasure RAS used in this case is not articulated but straight, so it must be moved between arms to allow an optimal working angle and reach. A second-generation articulated robotic Ligasure Maryland is in the pipeline but still not available on the market. In the present case, the use of Ligasure RAS allowed us to perform a fully robotic distal pancreatectomy. However, the manipulation of the straight instrument was tricky, required application through different arms at different stages and gave a certain laparoscopic flair to the robotic procedure, unfortunately without haptic feedback.

Some of the technical aspects of the procedure deserve to be addressed separately. Our choice of a lithotomy instead of prone position used by other authors may offer an advantage in case of conversion to laparoscopy and does not interfere with the movement of the robotic arms or the position of the bed assistant. An epigastric port is not usually used by us for upper-GI surgery with the Hugo; however, in this case, we had to position the ports in a line lower than usual to allow for a good reach over the whole upper abdomen. As Hugo instruments are about 10 cm shorter than Da Vinci’s ones, the access to the region of the spleen and uppermost short gastric vessels may be an issue; this is why we chose to have an additional port as high as possible for the first step of the procedure. During the second step, we used an 8-in-12 mm port-in-port technique, which is our preferred method to reduce the number of ports in colorectal surgery and works well with the Hugo arms. The robotic port was removed only for the three applications of the stapler through the 12 mm port. We also used two 11 mm ports to allow different endoscope positions and thus an optimal view of the surgical field. No Air-Seal access port was used and we never use it with Hugo; exact dissection in the correct anatomic plane and bloodless operative field results in minimal smoke production, which does not interfere with the surgical view. A liver retractor was also omitted in this case because of a relatively small left lobe and the intragastric approach. If a Nathanson retractor is needed, the insertion place of the epigastric port may be use; however, the arm carts of Hugo are quite bulky and an external retractor may easily interfere with their movement. This is why we prefer using intracorporeal liver retraction with sutures for robotic upper-GI surgery. Another specific feature of our technique is the intentional use of Hugo’s 0° endoscope in all robotic procedures, since it provides a predictable straightforward movement and prevents the carrying arm from being in extreme positions, thus reducing the risk of arm collisions. Alternatively, a 30° endoscope is available and may be used as a surgeon’s preference. Another major difference from the technique described by Nagai et al. is represented by the arm configuration: we stick to the “butterfly” universal setup for all Hugo RAS procedures, including this pancreatic resection. The 3-1 cart setup used by Nagai et al. is often used by a solid fraction of Hugo surgeons for various types of procedures to provide more space for the bed assistant. In our opinion, the 2-2 setup offers more freedom and variability for instrument allocation and causes fewer arm collisions. Moreover, in fully robotic DP, the bed assistant is rarely in the vicinity of the arms.

## 5. Conclusions

In conclusion, we successfully performed a fully robotic distal pancreatectomy with splenectomy with the Hugo RAS platform implementing the Ligasure RAS. To our knowledge, this is the first reported pancreatic resection using the Hugo platform from Europe and second published Hugo RAS RDP after that of Nagai et al. worldwide. As pancreatic surgery is complex and demanding, the regular use of the Hugo RAS platform for such procedures will strongly rely on the introduction of a wide spectrum of advanced robotic instruments at least analog to the ones of the DaVinci system. Until then, Hugo robotic-assisted surgery of the pancreas will remain an off-label use restricted to a small number of Hugo-experienced, high-volume pancreatic surgeons.

## Figures and Tables

**Table 1:** Timeline of key clinical and diagnostic events.

Time	Event	Major Findings
July 2023	CT chest/abdomen for episodes of upper back and lumbar pain	Aortic ulcer, incidental cystic lesion 10 mm in pancreatic body
February 2024	MRI/MRCP as part of wait and watch strategy, first diagnosis of NIDDM	Cystic lesion grown to 13 mm, first calcifications around it
December 2024	MRCP as part of wait and watch, no symptoms	Cystic lesion grown to 16 mm, more calcifications
August 2025	CT/MRCP, first episodes of postprandial pain, nausea, diarrhea	Cystic lesion grown to 20 mm, first solid components
February 2026	MRCP/EUS as preoperative evaluation, weight loss 10 kg since 08/2025	Cystic lesion grown to 22 mm, solid contrast enhancing wall

## Data Availability

The original contributions presented in this study are included in the article. Further inquiries can be directed to the corresponding author.

## Supplementary Materials

The following supporting information can be downloaded at: https://www.mdpi.com/article/10.3390/jcm15062423/s1.
